# Effect of electro‐activated brine solution on the migration of metallic ions from the cans to the product in sterilized canned sweet corn

**DOI:** 10.1002/fsn3.357

**Published:** 2016-03-24

**Authors:** Viacheslav Liato, Steve Labrie, Marzouk Benali, Mohammed Aider

**Affiliations:** ^1^ Institute of Nutrition and Functional Foods (INAF)Université LavalQuebecQuebecG1V 0A6Canada; ^2^ Department of Food SciencesUniversité LavalQuebecQuebecG1V 0A6Canada; ^3^ Natural Resources Canada/CanmetENERGY1615 Lionel‐Boulet Blvd.P.O. Box 4800QuebecVarennesJ3X1S6Canada; ^4^ Department of Soil Sciences and Agri‐Food EngineeringUniversité LavalQuebecQuebecG1V 0A6Canada

**Keywords:** Electro‐activation, food canning, metal migration

## Abstract

Tinplate cans were used to study if electro‐activated brine solution (EAS) is more corrosive than conventional one by ICP analysis. The results showed different effects of EAS on cans, alone or filled with product. Acidic EAS (pH 2–3) and Redox +900 to +1200 mV highly reacted with the cans. The concentrations of Zn, Fe, and Cu in the solution were 0.028, 28.81, and 0.022 ppm, respectively. No Sn migration was observed in this case. When neutral or acidic chlorine‐free EAS was used, no significant difference was observed in comparison with the corrosivity of standard NaCl brine. Alkaline EAS with pH>10 and negative E (≤−966 mV) did not affect Zn, Fe, and Cu migration. However, it affected tin migration. Nevertheless, it is important to mention that even if some corrosion was observed, it was in the limit of the permitted level of concentration when the cans were filled with a product.

## Introduction

Technology of electro‐activation is based on passing a direct electric current in the reactor cell through an aqueous solution (electrolyte) in which the redistribution of ions in an electric field simultaneously occurs along with the electro‐activation of molecules, atoms, and ions (Leonov et al. 1999). On one hand, during the anode electrochemical treatment, the acidity of solutions (anolyte) increases to pH ≈ 2, the oxidation reduction potential (E) increases up to +1200 mV; on the other hand, as a result of cathode treatment the solution (catholyte) becomes alkaline, its pH reaches ≥11 while the E is sharply reduced to −900 mV and below (Bakhir et al. 2002).

Research on the properties of electro‐activated solutions (EAS) has a wide array of applications in numerous domains (Gnatko et al. 2011). EAS have been utilized as a sanitizer with numerous advantages such as strong disinfecting property, easy operability, relatively inexpensive, and being environmentally friendly (Bakhir et al. 2002; Huang et al. 2008; Gnatko et al. 2011; Aider et al. 2012). By using this type of a sanitizer, the food industry may reduce microbial numbers to safe levels; however, it may have side effects such as corrosion of food contact surfaces because of its reactivity. Correct and thorough evaluation of the reactivity of sanitizers with respect to the equipment to which they will be applied is therefore extremely important (Ayebah and Hung 2005a).

Previously, several studies reported that different metals such as stainless steel, carbon steel, aluminum, copper, and dental alloys (Au–Ag–Pd and silver) exhibit high rate of corrosion, as measured by mass loss and surface roughness changes, in acidic media with low pH (<3) (Ayebah and Hung 2005a; Waters et al. 2014). Furthermore, pH was found to play important role in the corrosion intensity (Tanaka et al. 1999; Ayebah and Hung 2005b; Xia et al. 2012; Waters et al. 2014). Many studies have also determined that corrosion rates in chlorine‐based sanitizers depends on the chlorine concentration; especially for stainless steel, and that increased chloride concentrations lead to higher rates of corrosion (Waters et al. 2014). Although solution components such as chloride ion are an important factor that determine corrosion rates in chloride‐based sanitizers, its role in EAS is still not clear (Waters et al. 2014). It should be noted that there is a lack of information about corrosion and migration rates of the metal ions from the food containers (cans) in contact with EAS. It has been reported that internal corrosion of cans may increase the concentration of metal contents like Zn, Cu, Fe, Sn, and Al. There are significant problems associated with the use of tinplate cans in corrosive food products, such as corrosion failure, loss of seal integrity, or discoloration problems that result in their rejection by the consumer (Tuzen and Soylak 2007). Also, if this increase in heavy metals in the canned food exceeds the prescribed limits, they may become toxic to human health (Arvanitoyannis 1990).

Thus, in this work, we investigated the relaxation time and the corrosivity of EAS with respect to the type of metallic cans currently used for canning by the food industry.

## Materials and Methods

### Chemicals

Solutions were prepared by dissolving crystalline sodium chloride (NaCl) and sodium bicarbonate (NaHCO_3_) (Laboratory MAT, Montreal, Qc, Canada) in 1 L of distilled water to give a final concentration ranging between 0.5 to 0.05 mol/L. Sodium hydroxide (NaOH) and sodium chloride (NaCl) were purchased from VWR International LLC (West Chester, PA). Concentrated hydrochloric acid (HCl) was purchased from Fisher Scientific (Ottawa, Canada).

### Generation of EAS

In this study, a three‐cell electro‐activation reactor was used. The ruthenium–iridium‐coated titanium electrodes were connected to a direct electric current power source so that the anode was connected to the positive side and the cathode was connected to the negative side of the DC‐electric current generator (Lambda, GR.260; Electronics Corp., Melville, NY). The anode and cathode compartments were separated from the central compartment by an anion (AM‐40) and cation (CM‐40) exchange membranes which were purchased from Schekinaazot ltd., Schekino, Russia). The electrodes (100 × 40 mm) were placed in the anode and cathode compartments, with the distance between the electrodes being 150 mm.

The EAS was made from two configurations. In the first configuration, all the three compartments were filled with NaCl (0.05 mol/L); in the second configuration, the cathode and the central compartment were filled with the NaCl (0.1 mol/L) solution, whereas the anode compartment was filled with NaHCO_3_ (0.05 mol/L). The resulting EAS from both the configurations was optimized as described in previous work to give the pH value of each solution between 2 to 6. The solutions were named A2, A3, A4 (configuration #1), and A6 (configuration #2), the excitation time for each in the reactor was 30, 14, 5, and 60 min, respectively.

During the treatment EAS was analyzed for change in pH and redox‐potential (E) using a pH‐meter (Model SR 601C SympHony; VWR Scientific, Chicago, IL) and ORP‐meter (Eco Sense ORP15A; YSI Inc., Yellow Springs, OH). ZoBell's standard solution was used for the calibrating the ORP‐meter. The values of the applied voltage were measured with a Lambda generator (GR.260, Electronics Corp.,) and the intensity of the electric current was measured using a circuit‐test (DMR‐1000) which was connected to the power source.

### Relaxation period

Duplicate sets of EAS were generated and collected in 250 mL narrow mouth glass reagent bottles (Corning Pyrex, Corning, NY). The bottles were subsequently closed with glass stoppers and stored in dark at ambient temperature (22 ± 3°C). During the storage, the change in redox‐potential (E), pH, dissolved oxygen (DO), and total residual chlorine concentrations (RC) of the solutions were measured periodically. The dissolved oxygen was measured using a DO meter (Model SB 90M5 SympHony; VWR Scientific). Measurement of the redox‐potential and pH was done as detailed above. Total residual chlorine (free and combined chlorine) was measured with a chlorine colorimeter kit (Orion AQ3070, Thermo scientific AQUA‐fast, Singapore).

### Corrosion dynamics of tinplate can

For the corrosion analysis of food surface container, the enameled white can 300 × 407 mm (Dominion and Grimm, Quebec, Canada) was used. The tested solutions were prepared in the day of experiment, heated to 85 ± 5°C and seamed (Dixie UVG6MD, USA). The seamed cans were sterilized at 121°C during 20 min in a horizontal autoclave (Gebr.Stork & Co. In, Holland, Netherlands) without agitation, chilled, dried, and stored at ambient temperature (22 ± 3°C) (Platonova 2009). For analyzing the dissolution of the metal ions in the stored product, the targeted metal ions were analyzed and quantified by the Inductively Coupled Plasma method (ICP, Optima 4300 DV, Perkin‐Elmer, Norwalk, CT). The concentration of zinc (Zn), iron (Fe), copper (Cu), tin (Sn), and aluminum (Al) was measured with the wavelengths of 213.857, 239.562, 324.752, 283.998, and 237.313 nm, respectively.

### Statistical analysis

Each analysis was carried out in triplicate. Minitab software (V16.0, Minitab Inc. State College PA) was used for all statistical analysis of the obtained data, including evaluating the process efficiency or comparing the mean values. The Tukey's method (one‐way ANOVA) with a 95% confidence level was used.

## Results and Discussion

### Effect of relaxation time during storage, on EAS

Table 1 demonstrates the proprieties of EAS, acidified (NaCl‐2) and non‐acidified (NaCl) control solutions. The physicochemical properties of EAS depends on the characteristics of the electrochemical cell and its operating parameters (Liato et al. 2014). Thus, as seen from Table 1, configuration one generates solutions (A2, A3, and A4) with low pH and high (E) while the second configuration generates solution (A6) with neutral pH and high E. The EAS differed in their content of RC, D.O., E and pH.

**Table 1:** Properties of tested electro‐activated solutions

Solutions	pH	E, mV	RC, mg/L	DO, mg/L
A2	1.98 ± 0.12	>1200	205 ± 10	19 ± 3.5
A3	3.05 ± 0.10	1115 ± 11	34 ± 6	14 ± 6.1
A4	3.98 ± 0.11	915 ± 8	4 ± 2	11 ± 3.3
A6	6.45 ± 0.07	935 ± 7	350 ± 17	25 ± 2.2
NaCl	6.50 ± 0.04	355 ± 11	–	4.6 ± 1.8
NaCl‐2	2.00 ± 0.03	395 ± 10	–	4.5 ± 2.9

DO, dissolved oxygen; RC, residual chlorine.

**Figure 1:**
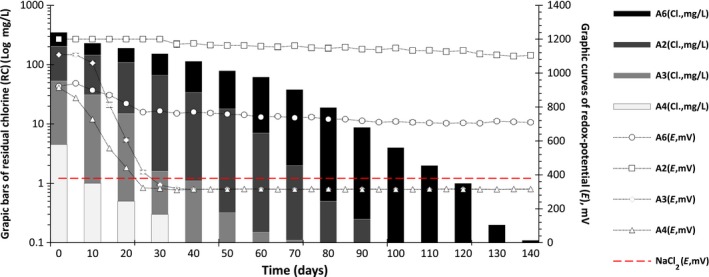
The effect of storage on the change of free residual chlorine (bars) and of redox potential (curves).

A number of studies involving analysis of the physicochemical and the antimicrobial properties of EAS during long‐term storage found that the best method of preserving the solutions is keeping it sealed in a chemically inert container, like a glass flask (Bordun and Ptashnyk 2012). During the analysis, the solutions A3 and A4 were found to be the most unstable; their redox potential decreased and equaled that of the control solution (NaCl‐2) at (380 ± 11 mV) at the end of the 30 days storage period (Fig. 1). Although concentration of DO in EAS was observed to have decreased to 4.6 ± 1.8 mg/L equal to that of the DO in the control solution (NaCl) after 5 h a significant change in the redox potential of EAS was not seen(data not shown). The concentration of the residual chlorine decreased below the detection level (0.02 mg/L) of A4 at 40th day and for A3 at 70th day, respectively. Our results were in accordance with those reported by Hsu and Kao (Hsu and Kao 2004) who suggested that EAS exposed to the atmosphere contained less chlorine and oxygen than when kept in a closed systems for a long time. The author pointed out that in their experiment, after 21 days of measurement (opened only for measurements), they observed a significant decrease in residual chlorine and dissolved oxygen in the EAS stored in open environment probably due to evaporation, which followed first‐order kinetics under open conditions, but no such decrease was observed under closed conditions. The solutions A2 and A6 showed the longest time of relaxation, and their redox potential was stable for more than 140 days (Fig. 1). It should be noted that the treatment (generation) time of A2 and A6 solutions was 30 and 60 min; markedly longer than others. Based on recent investigations, it may be assumed that the most activated form of EAS is obtained near the electrode interface, where a variety of reactions occur; therefore, it may be safely assumed that a longer excitation time may result in the generation of more metastable compounds (i.e., radicals, chlorine compounds, dissolved oxygen etc.) in the EAS (Prilutsky and Bakhir 1997). This assumption could also explain the longer relaxation period of the redox potential (E) of both EAS. The E values of A2 and A6 decreased to +377 mV after 190 and 270 days (data not shown). The concentration of residual chlorine (RC) of solutions A2 and A6 decreased gradually; by the 100th day, the RC concentration of A2 decreased below the detectable limit (Fig. 1), and the solution A6 showed the same result on 140th^ ^day, however, the E value of both solutions remained stable throughout the storage duration. A decrease in RC during the long‐term storage test of EAS was observed by Robinson et al. (2012), and they found that EAS stored for more than 200 days had a free chlorine concentration of ≤0.01 mg/L. They also found that the bactericidal activity did not change after the storage duration even in the absence of measurable chlorine. However, in the work of Len et al. (2001), it was seen that during the storage of EAS, a reduced bactericidal activity was observed possibly due to the evaporation of the dissolved chlorine gas and ensuing decomposition of hypochlorous acid (HClO). The main disadvantage of EAS is the decrease in its bactericidal activity after the relaxation period, and it reverting to an ordinary salt solution wherein the pH remains generally unchanged. Thus, it should be noted that pH could also be one of the important factors influencing the relaxation time of EAS. In our previous study, we showed that the variation in the pH of the EAS was not significant after a long relaxation time which was in good agreement with the work reported by Cui et al. (2009). They observed that pH of neutral and acidic EAS remained stable till the end of investigation. Furthermore, authors found that loss of available chlorine was significantly higher in acidic EAS than in neutral EAS.

Bakhir et al. suggest that the main disinfecting element in EAS might be the residual metastable chlorine; depending on the pH value of the EAS, the equilibrium reaction of chlorine may shift and result in an increased concentration of hypochlorite anions (ClO^−^) (Bakhir et al. 2003). The generation of chlorine depends upon a specific combination of E and pH where a minimum pH value of (<3) is required for chlorine gas (Cl_2_) production while a pH ≥3 results in production of only HClO and ClO^−^ (Pourbaix 1974; Zaviska et al. 2012). Therefore, a shift in this equilibrium may result in production of lesser Cl_2_, favoring formation of HClO, which is not volatile and could probably slow the decrease of redox potential as well as result in a longer relaxation time. A similar result was shown by Len et al. (2001), in their study involving EAS and chlorinated water with different pH values ranging between (6.0 and 9.0.); they reported that the solutions with lower pH displayed significantly higher loss of chlorine than solutions with alkaline pH. However, in a closed environment, as in this study, the primary mechanism of chlorine loss may be self‐decomposition of chlorine species in the solution (in the absence of evaporation).

### Effect of EAS on the corrosion of tinplate containers

**Figure 2:**
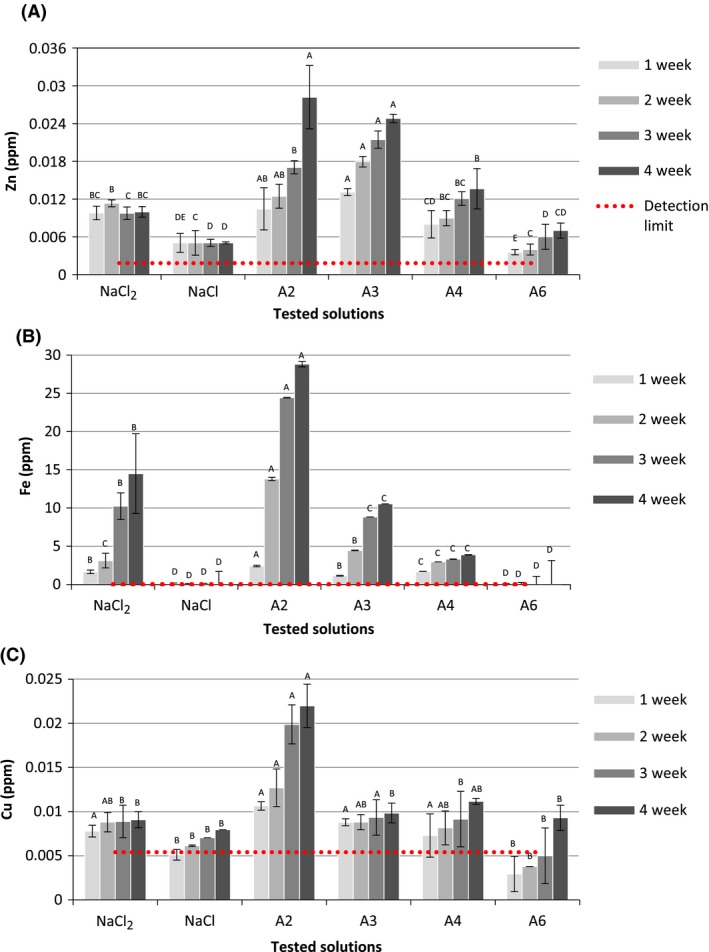
Changes in zinc (A), iron (B), and copper (C) concentrations during 4 weeks storage with different tested solutions.

The data obtained by inductively coupled plasma can help determine the concentration of the heavy metals into the solution that could have migrated from the container walls (Fig. 2). The concentration of zinc was more pronounced in solutions A2 and A3 (0.028 and 0.025 ppm) followed by A4 (0.014 ppm) and the acidified control solution (NaCl‐2) at 0.010 ppm (Fig. 2A). The solutions A6 and neutral control solution (NaCl) were found to be least corrosive with the dissolved Zn concentration being at 0.006 and 0.005 ppm, respectively. After 4 weeks of storage, the concentration of iron in solution A2 (28.814 ppm) was observed to be the highest and significantly different to that of the controls; NaCl‐2 (14.5 ppm) and NaCl (0.0061 ppm) (Fig. 2B). Solutions A3 and A4 with 6.992 and 3.883 ppm, did not have a significantly different concentration of iron to each other but were significantly different to the values of both the control solutions (*P* > 0.005). Furthermore, the neutral EAS (A6) 0.0062 ppm had the smallest concentration closest to the detection limit of 0.0018 ppm.

Figure 2C shows that in 1 month, the concentration of copper was low and close to the detection limit (0.0054 ppm) for all solutions except A2, which displayed a significant corrosion rate ability to corrode the container. The concentration of copper in A2 (0.0220 ppm) was twice as much than in the other samples. Even though the solutions A4, A3, A6, and control samples were less active, the presence of Zn indicated that they had caused corrosion (0.0112, 0.0098, 0.0091, 0.0089, and 0.0080 ppm, respectively). Corrosion may be defined as a process dependent on the interaction of a material and its immediate environment which results in the degradation of the material surface (Waters et al. 2014). The main characteristic of corrosion is the reaction rate which depends on the environmental impact (such as contact with water, acids, bases, salts, some chemicals, gaseous compounds like acid vapors, sulfur‐containing gasses, or ammonia gas) and time. Tinplate of cans is a light gage, cold reduced, low‐carbon steel sheet or strip, coated on both sides with pure tin. Many studies have shown that corrosion process of tinplate is complicated due to its scarified structure and heterogeneity. The corrosion of tinplate in contact with NaCl solutions increases significantly with an increase in salt concentration (Xia et al. 2012). In our study, all solutions were 0.05 mol/L NaCl concentration, except A6 solution which contained 0.05 mol/L NaHCO_3_. The control solutions were prepared from NaCl (0.05 mol/L) solution, than adjusted to necessary pH by 0.025 mol/L HCl or NaOH. The pH of the media may be significant, due to the complex behavior of tinplate. If the EAS are able to retain their original properties (pH and redox potential) for a long time (Fig. 1), it is possible that they could stay stable with chemical properties and levels as at the time of preparation. However, at the moment of opening, the EAS lost its activated form and reverted to the normal state with a pH equal to the initial pH before the commencement of storage (data not shown).

Low pH in itself, but especially below 5, seemed to be more effective (capable) in diluting metals (copper, iron, etc.). Thus, the advantage of tinplate lies here in the fact that it protects the steel of cans. Some metals like aluminum, tin, and zinc increase in corrosion rate when pH is above 9 (Xia et al. 2012). In this study, aluminum and tin was not detected in the solutions or their concentration was below the detection limit (0.0230 and 0.096 ppm, respectively). However, alkaline solution (catholyte, pH = 12 ± 0.02, E = −966 ± 13 mV) showed high tin and aluminum dilution (6 and 0.05 ppm, respectively) after 4 weeks of experiment (data not shown). The change in metal level in the solutions is shown in Figure 2, and it can be seen that a decrease in the metal concentration is observed with a decrease in the pH of the respective EAS. The Tukey`s test was used to show the difference between each tested solution during the same time period. The control solutions also followed the same trend, with the pH of the solution being inversely co‐related to the metal concentration in the solution. However, studies performed on the corrosion of food cans have demonstrated that even though pH has a significant impact, it is not the only factor, for example the test with different acids like citric, malic, or tartaric showed that the acids alone were less corrosive than the fruits (Hartwell 1951). In works of Platonova (2009), it was shown that there was dissolution of iron and zinc in tomato, grape, and apple juices with pH values between 2.8 and 3.3 stored in tinplate cans after 10 days storage. The dissolution of iron was almost the same in all the samples (5.5 ± 0.2 ppm), while the dissolution of tin was found as following: grape > apple > tomato (47.5, 22.0, 15.0 ppm, respectively). Despite of the fact that the influence of pH is significant, the impact of EA solutions is more important in terms of corrosion rate with the course of time. In order to avoid the tin and iron contamination, lacquers or different kinds of corrosion inhibitors can be used in order to protect the surface of the container (Nincˇevicˇ Grassino et al. 2009). Although corrosion can form under the layer of lacquer due to a variety of reasons such as improper uniform coating, damaged layer during manufacturing, and packaging or the influence of other physical or chemical factors (Platonova 2009). It is known that EAS inherently includes reactive components such as active chlorine and dissolved oxygen which may cause high corrosion. (Tanaka et al. (1999) found that EAS (pH 2.3–2.7, E = 1000 mV, and available chlorine 10–50 ppm) has a higher rate of dissolving stainless steel of hemodialysis equipment than 0.1% sodium hypochlorite solution (available chlorine is 1000 ppm). He also found that for EAS, the concentration of ferric ions on the 36th day of soaking was 0.6 mg/L while sodium hypochlorite solution was less than 0.05 mg/L. In the work of Ayebah and Hung (2005a), stainless steel showed outstanding corrosion resistance in EAS (pH = 2.42, E = 1077 mV, chlorine concentration 48.66 mg/L), deionized water (pH = 6.37, E = 584 mV, chlorine concentration 0 mg/L), chlorinated water (pH = 8.72, E = 656 mV, chlorine concentration 49.16 mg/L), modified EAS (pH = 6.12, E = 774 mV, chlorine concentration 50.39 mg/L). The highest mass loss of metals such as carbon steel, aluminum, and copper coupons (immersed in tested solutions for 22 h) was observed in EAS, suggesting that chloride ions could be one of the primary causes of corrosion. Authors found highly significant correlation between the decreasing concentration of chlorine and average weight loss. In our study, after the storage duration in cans, the EAS exhibited changes in chemical proprieties after the first week of experiment. After opening the container, no available free chlorine was found; the E value decreased and was observed to be 380 ± 13 mV. The pH, however, did not change; therefore, pH may be assumed to be a crucial factor causing corrosion of the tinplate in this study. The work of Waters (Waters et al. 2014) also confirmed that pH of chlorine‐based sanitizers is a significant factor in determining the corrosion rate of different types of metals used in a food processing environment. However, the high concentrations of metals in this study indicate that EAS solutions have a significant corrosive effect on certain types of tinplate, depending on the nature and efficacy of the inner coating used in the manufacturing process. The corrosion of metallic materials in contact with aggressive media involves a whole range of factors, which may act singly or jointly (Ayebah and Hung 2005a). The simultaneous reaction of chemical components of EAS at temperatures such as during sterilization and filling (90°C) could cause significant damage to the coating layer (lacquer, tin). After visual observation, the majority of cans were found to have initiation points of corrosion on the side seam and locale sites. These observations suggest that this type of corrosion is the typical pitting corrosion induced by the breaking of the passivation film or coatings. The presence of reactive elements (free chlorine, chlorine compounds or dissolved oxygen) could accelerate the kinetics of reaction which can explain the significant difference in the corrosivity of acid EAS, neutral EAS and control solutions. In this study, the analysis of corrosiveness of EAS on the tinplate surface was carried out based on the indirect method of observing the migration of metals from the container to the solution, and additional studies are required to understand the reactivity of chlorine‐based solutions such as EAS in contact with the can surface in combination with different storage temperatures in as well as with different foods.

### Changing of metals concentration in the canned corn produced with EAS

**Figure 3:**
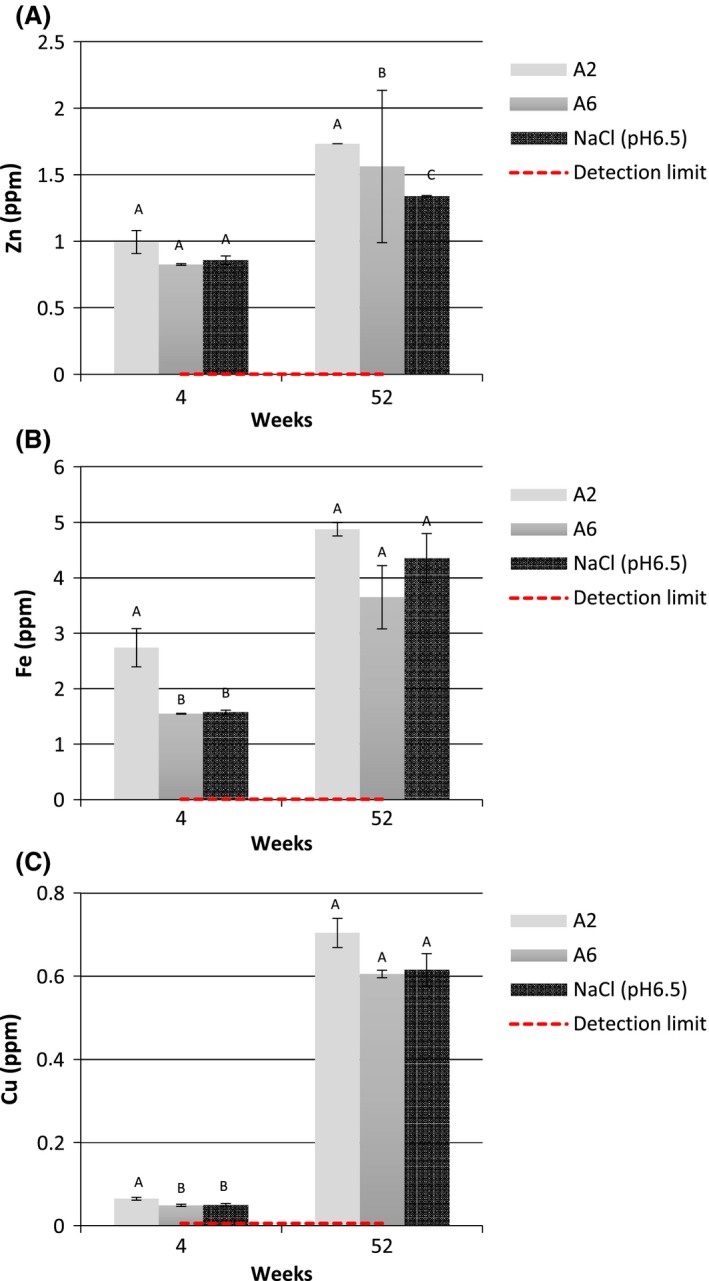
Changes in zinc (A), iron (B), and copper (C) concentrations during four and 52 weeks of storage in canned corn with different tested solutions.

The metal content found in canned corn is presented in the Figure 3. The Tukey's test was used to show the difference between the analyzed samples at one period of time. The corn samples were purchased from local market, then peeled, blanched, and sterilized as described above. At week 4 and week 52, analyses were carried out. After opening the container, no visual defects on surface was found but the increase in concentration of metal content with time indicates that the corrosion took place. The presence of aluminum and tin was not detected or their concentration was below the limit detection. At the end of the experiment, the highest level of metal was found in A2, where the concentration of zinc, iron, and copper were at 1.73 ± 0.0009, 4.87 ± 0.12, and 0.70 ± 0.04 ppm, respectively. It should be noted that the canned corn in EAS had a significantly low concentrations of the different metals, well within the safe consumption limit for tin (≥14 mg/kg, Codex, 1998), for zinc between 9–14 mg/day (NRC 2000), for iron 8.7–14.8 mg/day (Food Standard Agency 2010), copper 2.1–3 mg/day (WHO), and aluminum at ≥50 mg/day (WHO).

After 1 month of storage of the product in the EAS, a change in the properties of EAS was observed because after contact with any organic matter EAS becomes ordinary water (Huang et al. 2008). It was observed that A2 had significant difference in iron and copper content after 1 month and of zinc after 1 year. The synergy between different factors (chlorine ions, acidity and high redox potential) could cause corrosion, especially when the hot solutions come in contact with the can surface. Besides, the metal concentration detected partly could be due to their transfer from the product. The metal level in canned vegetables may vary in a large spectrum, for example the zinc, iron, and copper level have been reported in the range of 1.0–8.9, 9.3–76.0 l, and 0.07–7.30 ppm, respectively, significantly higher than found in our study (Tuzen and Soylak 2007). The influence of added materials (sugar, sirups, salt, etc.) or those present in vegetable substrate (amino or organic acids, phenols, etc.) may also significantly impact the corrosion rate, but these organic and inorganic substrates may also retard it (Hartwell 1951). Furthermore, the usual pH of the media is neutral, and the corrosion rate and related properties in such a condition are still uncertain (Xia et al. 2012). Corrosion in canned food may also be supported by the trace amounts of oxygen present due to subpar commercial practice or leakage, which has been recognized as a factor responsible for accelerating corrosion (Hartwell 1951).

## Conclusions

The relaxation time was longer for neutral EAS compared to that of acid EAS. The decrease in redox potential as a function of pH showed that neutral medium inhibits the decay of free chlorine in a relatively acidic medium it is accelerated. A better understanding between the relaxation time and the change in values of redox potential is important to clarify the reactivity of EAS during storage and more importantly the nature and attributes of electro‐activation of solutions. Contrary to assumption, it was found that temperature close to the boiling point did not weaken the EAS but accelerated its reactivity. Storage in glass bottle provided a good protective and inert environment for the EAS.

This study also showed that corrosion caused by free chlorine is not significant in EAS with pH near to neutral while in acidic EAS, the rate of corrosion was higher which may be attributed to the combined effect of pH and chemical compounds (free or/and combined chlorine, radicals etc.) present in EAS. Although EAS was found to be significantly more corrosive than the control solutions (in the canned corn and tinplate cans), the dissolution of metals in canned corn with EAS (neutral and acid) was equal to or lower than in canned vegetable food, as it was found in the control solutions. Therefore, EAS may be used as a noncorrosive replacement to the solutions used which in itself are primary agents of corrosion in canned food. Thus, to sum up, EAS is a viable alternative with its corrosiveness equal to or less than the canning solutions currently used in the food canning industry.

## Conflict of Interest

None declared.
